# Thermal tuning of protein hydration in a hyperthermophilic enzyme

**DOI:** 10.3389/fmolb.2022.1037445

**Published:** 2022-11-28

**Authors:** Giuliana Fusco, Carmen Biancaniello, Michail D. Vrettas, Alfonso De Simone

**Affiliations:** ^1^ Department of Chemistry, University of Cambridge, Cambridge, United Kingdom; ^2^ Department of Pharmacy, University of Naples “Federico II”, Naples, Italy

**Keywords:** protein hydration shells, thermal adaptation, hyperthermophilic proteins, water structure, water dynamics, restrained MD simulations

## Abstract

Water at the protein surface is an active biological molecule that plays a critical role in many functional processes. Using NMR-restrained MD simulations, we here addressed how protein hydration is tuned at high biological temperatures by analysing homologous acylphosphatase enzymes (AcP) possessing similar structure and dynamics under very different thermal conditions. We found that the hyperthermophilic *Sso AcP* at 80°C interacts with a lower number of structured waters in the first hydration shell than its human homologous *mt AcP* at 37°C. Overall, the structural and dynamical properties of waters at the surface of the two enzymes resulted similar in the first hydration shell, including solvent molecules residing in the active site. By contrast the dynamical content of water molecules in the second hydration shell was found to diverge, with higher mobility observed in *Sso AcP* at 80°C. Taken together the results delineate the subtle differences in the hydration properties of *mt AcP* and *Sso AcP*, and indicate that the concept of corresponding states with equivalent dynamics in homologous mesophilic and hyperthermophylic proteins should be extended to the first hydration shell.

## Introduction

As proteins evolve in aqueous environment, the protein-water interaction is one of the driving factors in the selection of native sequences able to fold and be functional under physiological conditions (Bianco and Franzese, 2015). Through the characterization of protein structures, it has now become evident that water is not simply the solvent of life but can also be an active component of proteins (Ball, 2017). Numerous studies have clarified the biological role of waters, including the stabilization of protein structures (De Simone et al., 2008), the modulation of protein-protein interactions (Vitagliano et al., 2011) or protein-ligand affinity (Qu et al., 2019), enzymatic catalysis (Pocker, 2000) and proton transport (Umena et al., 2011).

The hydration shells are also crucial modulators of the structural fluctuations of proteins, as indeed experimental and theoretical studies have shown that the dynamical behavior of proteins and of the surrounding waters are coupled (Tournier et al., 2003; Frauenfelder et al., 2006; Bianco et al., 2012; Bellissent-Funel et al., 2016; Rahaman et al., 2017). It was indeed proposed that protein dynamics require a fully connected hydrogen-bonded network of water molecules, activating the collective dynamics in a two-dimensional percolation transition (Oleinikova et al., 2005; Smolin et al., 2005; Mazza et al., 2011). In this context, an intriguing question is how proteins from hyperthermophilic organisms are able to attain physiological structural dynamics that are similar to those of their mesophilic homologues (Zavodszky et al., 1998; Radestock and Gohlke, 2011; Fields et al., 2015; Stirnemann and Sterpone, 2017) despite the remarkable differences in the translational-rotational kinetic properties of bulk waters at the respective physiological temperatures (Sterpone et al., 2010; Chakraborty et al., 2015; Rahaman et al., 2015; Krah et al., 2020). Hyperthermophilic proteins indeed can remain folded and active under conditions that often approach the water boiling temperature, posing the question on what are their hydration properties and how these contribute to achieve protein structural dynamics that are equivalent to those of mesophilic homologous.

In order to investigate the nature of protein hydration at high temperatures, we here used MD simulations restrained with experimental NMR chemical shifts (CS) to sample the nanosecond dynamics of two proteins from mesophilic and hyperthermophilic organisms, namely the human muscle acylphosphatase (*mt AcP*) and its homologue from *Sulfolobus solfataricus* (*Sso AcP*). The simulations were run in explicit solvent and were restrained using experimental CS measured respectively at 37°C and 80°C for *mt AcP* and *Sso AcP*. The results showed that, despite the significant difference in the native temperatures, the extension of the first and second hydration shells from the protein surfaces overlap in the two proteins, however, in *Sso AcP* at 80°C the water population of the first hydration shell was significantly reduced. Moreover, while the translational dynamics in the first hydration shells of the two proteins were found to be comparable, the mobility of waters in the second hydration shell was significantly higher in *Sso Ac*P at 80°C. Overall, these results describe how the coupling between protein dynamics and hydration is tuned at very different biological temperatures in AcP enzymes.

## Materials and methods

### NMR spectra assignment of *mt AcP* at 37°C

The assignment of NMR CS of *mt AcP* was performed at 37°C in 30 mM MOPS buffer at pH 7.0 (Fusco et al., 2022). The protein net charge under these conditions is +4.9. NMR samples were prepared by diluting lyophilised protein into 500 μl of 10% deuterated solution up to a protein concentration of 200 μM. Assignments of the NMR resonances of the backbone atoms of *mt AcP* were adapted from our previous work (Fusco et al., 2012) by measuring a series of 3D NMR spectra under the present experimental conditions. All NMR data were processed and analysed using TopSpin (Bruker BioSpin), NMRPipe and Sparky software packages.

### NMR spectra assignment of *Sso AcP* at 80°C

The assignment of NMR CS of *Sso AcP* at 80°C was performed in 30 mM MES buffer and pH 6.5, which mimics the physiological conditions of the protein. At this pH the net charge of the protein is expected to be −0.5. NMR resonance assignments of *Sso AcP* at 80°C were performed using a combination of 3D spectra (HNCA, CBCAcoNH, HNCACB, HNCO, HNcaCO, and HNHA) in conjunction with the assignment performed at 25°C (BMRB, entry code: 6398) (Corazza et al., 2006). It is worth noting that in our study, the truncated form of *Sso AcP* was employed (ΔN11), to include only the ferrodoxin-like domain as in *mt AcP*.

### Chemical shifts-restrained MD simulations

In this study, we employed CS to restrain MD simulations using our previously developed method NapShift (https://github.com/vrettasm/NapShift), which is based on artificial neuronal networks to model CS from structure and enable derivatives to apply experimental restraints in MD simulations (Qi et al., 2022). The CS restraints of NapShift are based on experimental CS of six protein atoms (Cα, Cβ, C’, N, HN, and Hα) and act on dihedral angles of the main chain (φ, ψ) and of the side chains (χ_1_, χ_2_) (Qi et al., 2022). NapShift restraints were implemented in the GROMACS package for MD simulations (Páll et al., 2020) and imposed by adding an experimentally driven energy term to the standard force field (Eq. 1),

$$V^{Total}=V^{FF}+V^{CS}$$ (1)

where the experimental term was modelled as a harmonic potential based on the calculated CS value (Eq. 2).

$$V^{CS}=K\overset{Nres}{\underset{i}{\sum }}\overset{6}{\underset{j}{\sum }}{\left(\delta_{ij}^{\exp}-\delta_{ij}^{calc}\right)}^{2}$$ (2)

The harmonic restraints were applied as flat-bottom potentials where the restraining force is zero when the difference between experimental and calculated CS values falls within the experimental error of the measurement.

Initial 10 ns equilibration simulations were performed starting from the NMR structures of *mt AcP* (Motamedi-Shad et al., 2012) and *Sso AcP* (Corazza et al., 2006), respectively at 310 K and 353 K by using the CHARMM36 force field (Huang and MacKerell, 2013) and TIP3P explicit water model (Jorgensen et al., 1983) and increasing the weight K of the restraint energy with respect to the empirical force field from zero up to a maximum value of 300 kJ•mol^−1^•ppm^−2^. The weight K was subsequently maintained constant during the equilibrium simulations. The incidence of the restraining forces at equilibrium was estimated in average to be 5% of the overall forces applied on backbone atoms. Equilibrium MD were run for 100 ns in the NPT ensemble by weak-coupling of the pressure and temperature with external baths. Temperature coupling was performed with the V-rescale method (Bussi et al., 2007) using a coupling constant of 0.1 ps and reference temperatures of 310 K for mt AcP and 353 K for *Sso AcP*. The pressure of 1 atm was coupled using the Berendsen method (Berendsen et al., 1984), with a compressibility value of 4.5 × 10^−5^ bar^−1^. Electrostatic interactions were treated using the particle mesh Ewald method (Darden et al., 1993). The integration step for the simulations was 2 fs and the restraints were applied at each integration step. All MD simulations were carried out using periodic boundary conditions and adopting LINCS as a constraint algorithm.

### Hydration analysis

Water density function around the protein surface was calculated using the positions of the oxygen atoms (De Simone et al., 2006). Each frame of the MD simulation was superimposed onto a reference structure by using the Cα atoms of the protein. The Cartesian coordinates of the water oxygen atoms were then used to calculate the water population in a discrete 3D grid of 0.5 Å of step size, by applying boundary conditions. Water density in each node of the 3D grid was normalised on the bulk density value, which was estimated in a shell extending from 8.0 to 10.0 Å from the protein surface.

The dynamics of the water in the MDHS was calculated using a time autocorrelation function (Eq. 3) to estimate the residence time in the MDHS.

$$P\left(\tau \right)=\sum_{\tau }\delta (W\left(t\right),W(t+\tau ))$$ (3)

where the delta function δ(W(t), W(t + τ)) assigns one if the same water occupies the hydration site at times t and t + τ. The resulting time-autocorrelation functions were fitted using a single exponential model to provide the residence time.

## Results

In order to generate new understanding of the protein-water interaction in native conditions, we compared the hydration properties of two homologous folded enzymes whose physiological temperatures differ of 43°C. In particular, we chose two acylphosphatases sharing limited sequence identity (25%) but possessing the same ferrodoxin-like fold topology (Stefani et al., 1997), the same pattern of secondary structure elements (5 β-strands and 2 α-helices in βαββαββ arrangement), and solving the same hydrolase function *via* the catalytic residues Arg 23 and Asn 41. The significant structural similarity of the two enzymes is also reflected in similar radii of gyration (12.8 Å for *mt AcP* and 12.1 Å for *Sso AcP*) and solvent accessible surface area (61.9 nm^2^ for *mt AcP* and 59.1 nm^2^ for *Sso AcP*). Having evolved in very different environments, however, the melting temperatures of the two AcP are considerably different (56°C for *mt AcP* and 100°C for *Sso AcP*) (Corazza et al., 2006), thus enabling the hyperthermophilic *Sso AcP* to be folded and functional at 80°C, a temperature where *mt AcP* is partially unfolded and mostly inactive. Overall, these characteristics make *mt AcP* and *Sso AcP* ideal homologous systems to elucidate the differences in the native protein hydration at different temperatures. As water equilibrates in the picosecond timescale, we here generated MD trajectories of 100 ns for each system to provide robust statistics about the structure and dynamics of the hydration shells. Moreover, the availability of experimental CS enabled to perform accurate simulations of the two proteins at the physiological temperatures, as in our study CS restraints improve the accuracy of protein force fields, which are not parameterised to reproduce protein dynamics at high temperatures.

**FIGURE 1:**
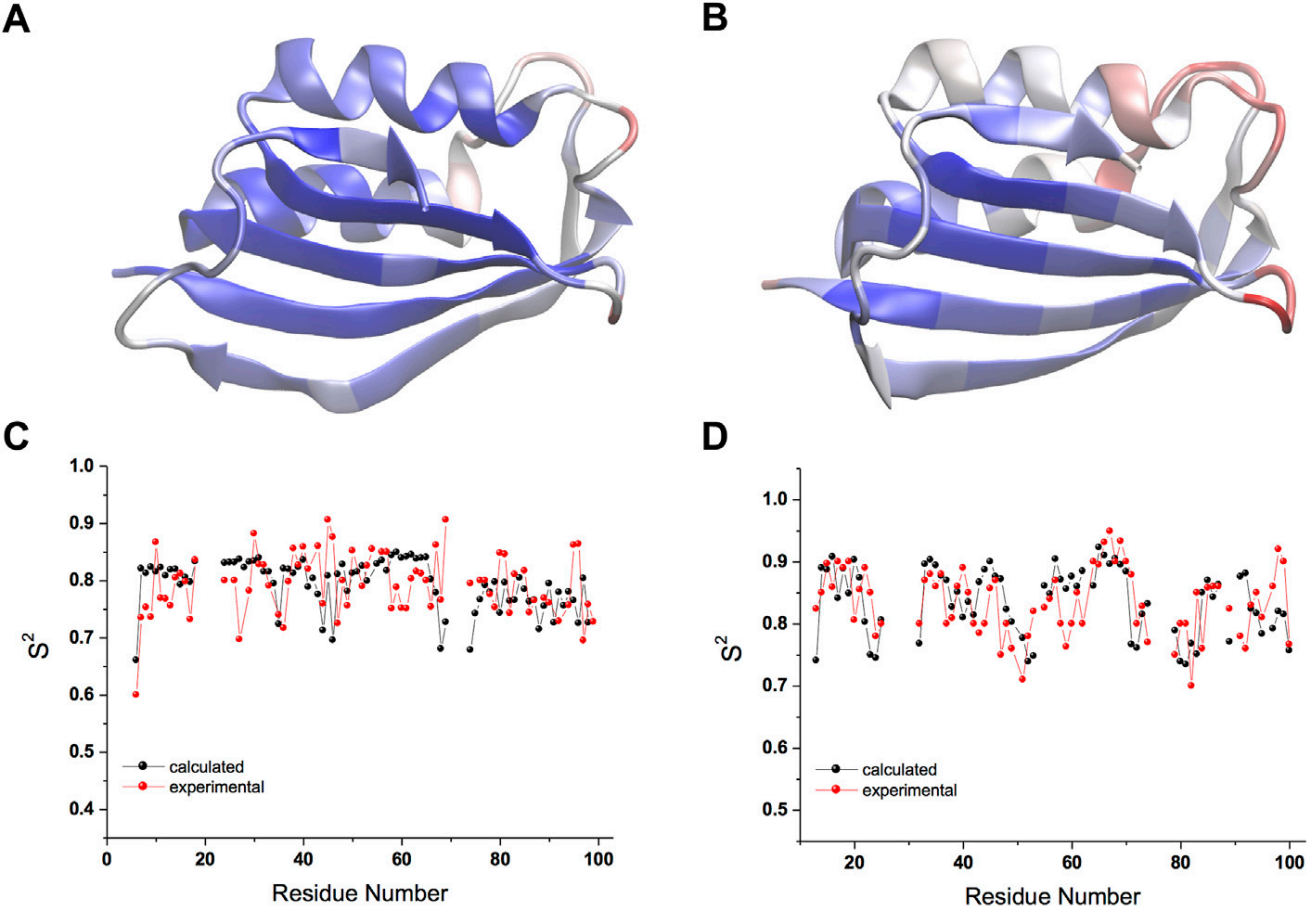
Nanosecond structural dynamics of *mt AcP* at 37°C and *Sso AcP* at 80°C. **(A,B)** root mean square fluctuations (RMSF) in the CS-restrained MD simulations of *mt AcP* at 37°C **(A)** and *Sso AcP* at 80°C **(B)** plotted onto the protein structures. Color code range from low (blue) to high (red) RMSF values. The corresponding RMSF graph is shown in Supplementary Figure S3. **(C,D)** Order parameters S^2^ values of *mt AcP* at 37°C **(C)** and *Sso AcP* at 80°C **(D)** calculated from the CS-restrained MD (black) and NMR data (red) from ^15^N relaxation measurements Fusco et al. (2022).

The CS-restrained MD trajectories of *mt AcP* at 37°C and *Sso AcP* at 80°C showed a significant agreement with experimental chemical shifts (Supplementary Figures S1, S2), as calculated using the SPARTA+ program (Shen and Bax, 2010), which is different from the NapShift method employed for the CS restraints in our MD samplings (Qi et al., 2022). The backbone dynamics of the two proteins in the trajectories showed similar patterns of rigid and dynamical regions, with the strongest fluctuations found in the loops connecting secondary structure elements (S1-H1, S2-S3 and H2-S4) as probed by root mean square fluctuations (RMSF, Figures 1A,B; Supplementary Figures S3A,B). The restrained MD samplings were also found to be in significant agreement with experimental order parameters S^2^ from ^15^N relaxation NMR (Fusco et al., 2022), which are sensitive probes of nanosecond dynamics in proteins (Figures 1C,D). Overall, the structures of the two proteins remained within Cα-RMSD values of 0.2 nm from the starting conformations, suggesting no significant rearrangements of the main chain in the simulations (Supplementary Figures S3C,D).

**FIGURE 2:**
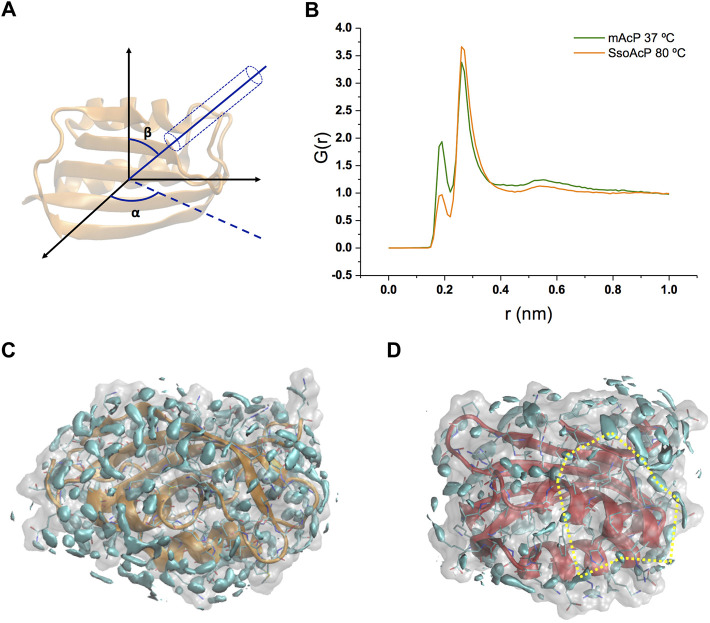
Structural properties of waters in the hydration shells of *mt AcP* and *Sso AcP*. **(A)** Schematic representation of the water radial distribution function G(r) calculation. A cylinder of 4 Å diameter was oriented by scanning the two Eulerian angles, *α* and *β*, with a step size of 20°. For each water molecule in the cylinder, the distance from the protein surface was used to calculate the G(r). The function was averaged across the values of *α* and *β*, and the simulation frames. **(B)** G(r) of *mt AcP* (orange) and *Sso AcP* (green). **(C,D)** Water density maps of *mt AcP* at 37°C **(C)** and *Sso AcP* at 80°C **(D)** contoured by cyan surfaces enclosing regions with a value of water density 2.0 times higher than the bulk average density. The density maps provide the location of the MDHS around the protein surface, which are defined as local maxima of the function in a radius of 0.14 nm, following the restriction to possess a minimum density of 2.0 times the value of the bulk water. In *SsoAcP*, large surfaces lacking MDHS were observed. One of these regions is highlighted using dotted yellow lines as an example.

Using these simulations, we analysed the structure and dynamics of water molecules around the two AcP enzymes at the respective physiological temperatures. As the structure and relaxation properties of bulk water are considerably different at 37°C and at 80°C, our study aimed at addressing the fundamental question of what is the protein-water interface at these temperatures in systems that have very similar structure and attain consistent backbone dynamics across multiple timescales (Fusco et al., 2022). As first, we calculated the water radial distribution function G(r) for the two enzymes. G(r) was averaged across the MD trajectories and calculated from the position of water molecules residing in a virtual cylinder positioned in the centre of mass of the protein and oriented in all possible combinations of the Eulerian angles *α* and *β* (using a step size of 20°, Figure 2A). The analysis indicates that, while the first and second hydration shells in the two proteins have essentially the same distance from the protein surface [G(r) maxima at 1.9 Å and 2.6 Å, Figure 2B], the population of the first peak is substantially depleted in *Sso AcP* at 80°C compared to *mt AcP* at 37°C. To obtain a more detailed analysis the structural properties of waters surrounding the proteins, we calculated the three-dimensional water density map, providing the position of the MD hydration sites (MDHS) that represent stably bound waters on the protein surface (Figures 2C,D). MDHS were assigned as the local maxima in the water density function in a radius of 0.14 nm, following the restriction to possess a minimum density of 2.0 times the value of the bulk water. We observed 60 and 35 MDHS in the first hydration shell of *mt AcP* at 37°C and *Sso AcP* at 80°C, thereby confirming the lower population of stably structured waters in the first hydration shell of the hyperthermophilic protein as observed in the G(r). Upon inspection of the residues in direct contact with the oxygen atoms of the waters, we found that the two enzymes have similar amounts of hyper-hydrated residues exposing charged side chains on the protein surface, however, the mesophilic *mt AcP* features a higher abundance of polar residues, particularly serine and threonine amino acids, in contact with the solvent (Supplementary Figure S4). This difference is reflected in the average number of waters surrounding the residues, respectively resulting in 0.53 and 0.43 for *mt AcP* at 37°C and *Sso AcP* at 80°C. When analysing the second hydration shell, however, the difference between the number of MDHS in *mt AcP* at 37°C and *Sso AcP* at 80°C was largely reduced (76 and 60 MDHS, respectively), in line with the similarity in the population of the second peak of the G(r) of the two proteins.

**FIGURE 3:**
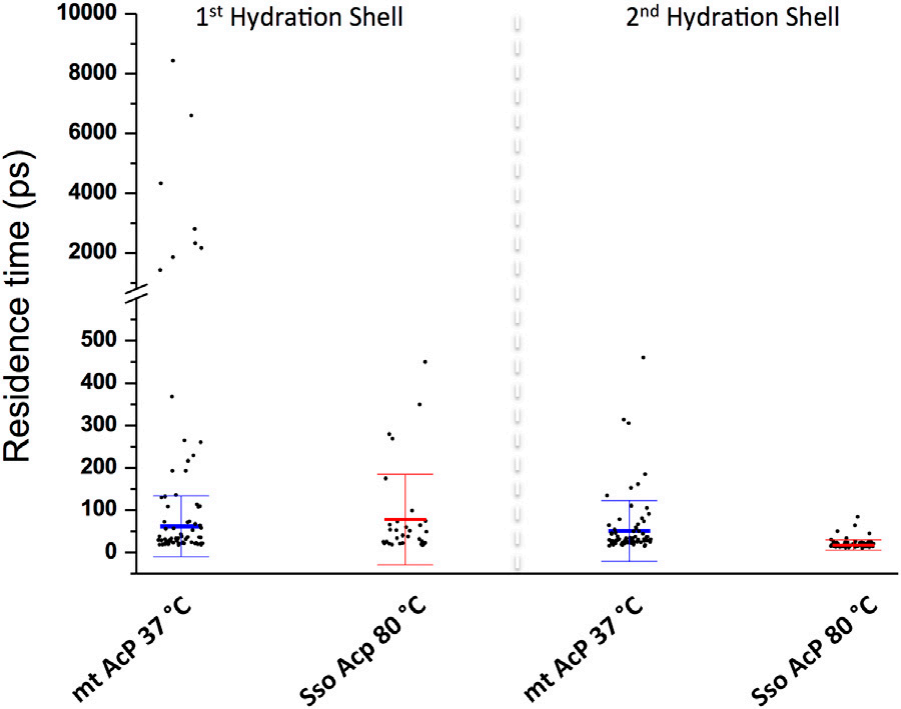
Dynamic properties of waters in the MDHS of mt AcP and Sso AcP. Water residence time in each MDHS was calculated using the autocorrelation function and a single exponential decay fitting model. The MDHS were classified as those belonging to the first or second hydration shell. Scatter plots of the residence times are reported for the two classes of MDHS for *mt AcP* at 37°C and *Sso AcP* at 80°C. In the first hydration shell, the average residence times were calculated by excluding superstructured MDHS (residence times higher than 1.0 ns), resulting in 62.5 (±72.2) ps and 78.5 (±99.7) ps for *mt AcP* at 37°C and *Sso AcP* at 80°C, respectively. In the second hydration shell, average residence times were 51.6 (±72.2) ps and 17.9 (±12.1) ps for *mt AcP* at 37°C and *Sso AcP* at 80°C.

**FIGURE 4:**
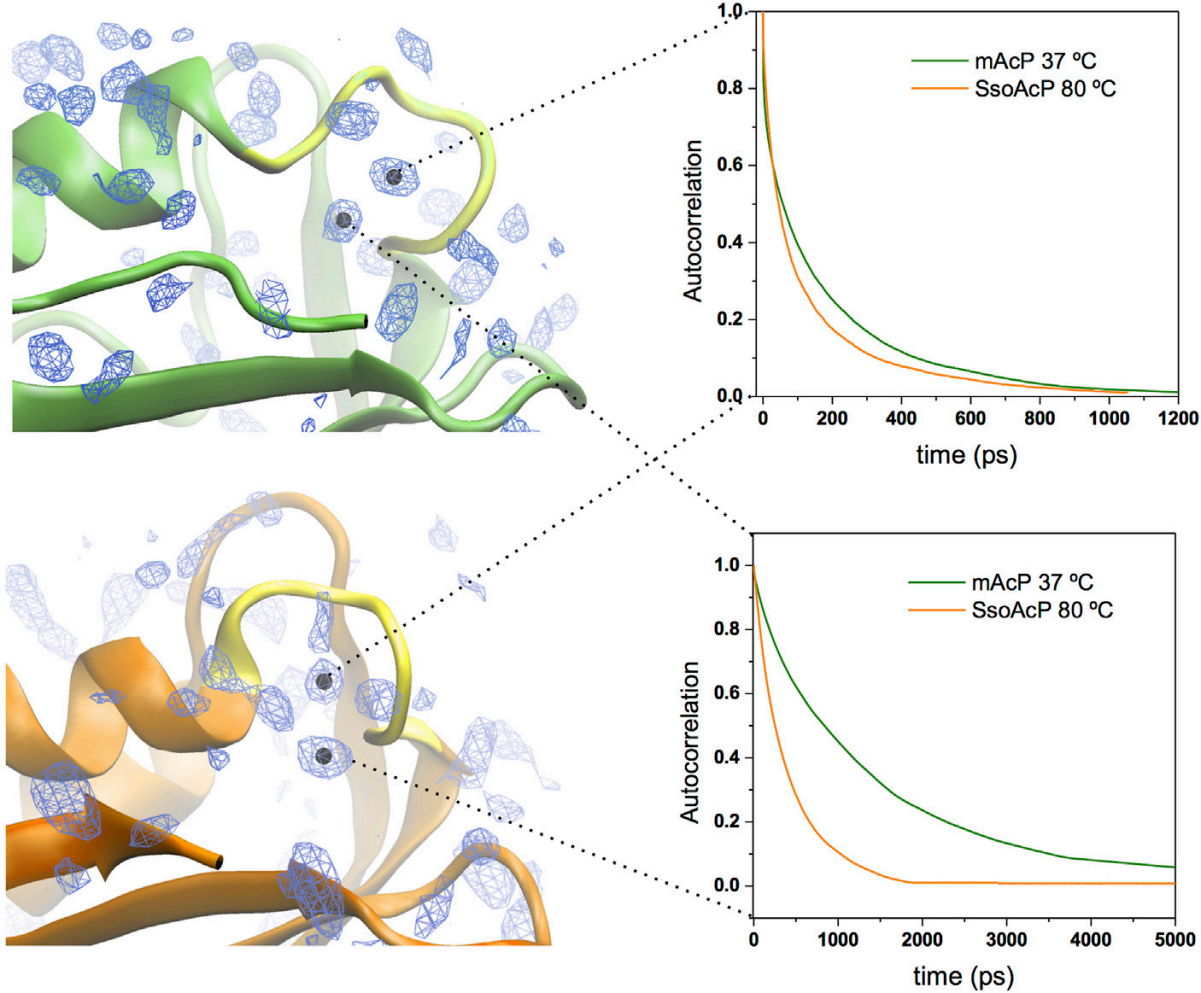
Structured waters in the active site of *mt AcP* and *Sso AcP*. Two conserved MDHS were found in close contact with the phosphate binding loop in both *mt AcP* and *Sso AcP*. Water density map is shown as blue contour lines with the two conserved MDHS marked with black dots in the centre. *mt AcP* (top) and *Sso AcP* (bottom) are rendered as green and orange ribbons, respectively, with the phosphate binding loop rendered in yellow for both systems. Autocorrelation functions of the waters in two MDHS are shown in the panels on the right (green and orange lines for *mt AcP* and *Sso AcP*, respectively). The resulting residence times are 311.5 ps (*mt AcP*) and 346.9 ps (*Sso AcP*) for the water in direct contact with the phosphate binding loop (top panel) and 4,297.5 ps (*mt AcP*) and 277.9 ps (*Sso AcP*) for the water interacting with Asn 21 (bottom panel) and participating to the catalytic mechanism Stefani et al. (1997).

We then characterised the dynamical behaviour of waters at the protein surface by calculating the water residence time in the MDHS. The data indicated that the translational dynamics of waters residing in the first hydration shell is similar in the two proteins, with average values of 62.5 ps and 78.5 ps for *mt AcP* at 37°C and *Sso AcP* at 80°C, respectively (Figure 3). When analysing the dynamics of hydrogen-bonds established between the proteins and the surrounding waters, however, we found a longer lifetime in *mt AcP* than in Sso AcP (137.9 ps vs. 58.7 ps, Supplementary Figure S5). In addition, by closely inspecting the properties of waters in the first hydration shell, we also observed two MDHS in proximity of the phosphate binding loop that are conserved in both proteins (Figure 4). These MDHS correspond to bound waters found also in the crystal structures of *Sso AcP* and acylphosphatases from other organisms (Supplementary Figure S6). In both proteins, the water residence times in these hydration sites were found to be significantly longer than the average residence time of MDHS in the first hydration shell. More specifically, a first water establishing direct hydrogen-bonds with the backbone amides of the phosphate binding loop was found to have residence times of 311.5 and 346.9 in *mt AcP* and *Sso AcP*, respectively. A second water, which directly interacts with Asn 21 and is known to participate to the catalytic mechanism of the AcP (Stefani et al., 1997), was found to have residence times of 4,297.5 ps and 277.9 ps in *mt AcP* and *Sso AcP*, respectively. While the latter residence times are different, they are both significantly higher than the average values of the first hydration shells of the two proteins. These data indicate that the hyperthermophilic AcP maintains structured waters in the catalytic site with long residence times in analogy to its mesophilic homologue. Thus, despite the considerable difference in the properties of the bulk water at 37°C and 80°C, structured waters in catalytic pockets behave in a similar manner.

Finally, when analysing the second hydration shells of the two proteins, we found that residence times in the hyperthermophilic system are significantly shorter than those of the mesophilic homologue, with average values of 17.9 ps and 51.6 ps for *mt AcP* at 37°C and *Sso AcP* at 80°C, respectively. Taken together these data indicate that the dynamical properties of waters in the first hydration shells of *mt AcP* and *Sso Ac*P are similar, and begin to diverge in the second hydration shell, where faster dynamics were observed in *Sso AcP* at 80°C.

## Discussion

Understanding the behaviour of water molecules at the protein surface is of paramount importance for elucidating fundamental mechanisms in biochemistry. In addition to playing a primary role for the hydrophobic effect (Niether et al., 2018), a major driving factor for protein folding, waters can participate explicitly or implicitly to the biological function of proteins (Ball, 2008). Tightly bound waters adopting stable three-dimensional positions in the protein structure have been associated with a variety of functional roles, such as the stabilization of the collagen triple helix (De Simone et al., 2008), catalytic reactions, the mediation of ligand binding (Tame et al., 1994) and the determination of ligand selectivity and specificity (Qu et al., 2019). A more enigmatic role is played by mobile waters, which can influence protein function in an indirect manner. Highly dynamical and poorly structured waters can indeed drive protein-protein interactions by defining dewetting-prone surfaces that reduce the cost of generating dry interfaces upon formation of macromolecular complexes (Vitagliano et al., 2011) or during protein aggregation into amyloids (Colombo et al., 2008). The dynamics of waters at the protein surface is also directly connected with structural fluctuations of the backbone and side chain atoms of proteins. Numerous studies have indeed indicated the presence of a coupling between protein dynamics and hydration shell dynamics, suggesting that the dynamical behaviour of the hydration shell is a reservoir of fluctuations that favour conformational transitions in proteins (Ball, 2017). This coupling has also been observed in allosteric conformational transitions (Buchli et al., 2013), whereas emerging theories suggest that protein dynamics are indeed “slaved” by the hydration shell in small systems and by the bulk solvent in large systems (Frauenfelder et al., 2009).

In this context, a fascinating question arises as to how protein dynamics and hydration dynamics are coupled in systems evolutionary optimised to work at very high biological temperatures. In particular, a fundamental debate is how waters at the interface of hyperthermophilic proteins behave at high temperatures while these proteins maintain similar structure and dynamics of their mesophilic homologues despite the bulk solvent possesses considerably high kinetic energy. We here aimed at answering this question by using MD simulations to sample the hydration properties of two homologous acylphosphatase enzymes adopting very similar structures and solving the same function at very different temperatures. Using extensive NMR analyses, we previously showed that the backbone dynamics of *mt AcP* at 37°C and *Sso AcP* at 80°C are similar across a range of timescales (Fusco et al., 2022). We now used CS-restrained data to overcome possible biases in protein force fields, as these are not specifically parameterised to reproduce protein dynamics at high temperatures. The trajectories enabled to analyse the structure and dynamics of the hydration shells of the AcP enzymes at 37°C and 80°C, showing no major differences in the positions of the first and second hydration shells with respect to the protein surfaces. The population of the first hydration shell in *Sso AcP*, however, was found to be nearly half of that observed in *mt AcP*, a finding that is in line with a reduced number of MDHS in the first hydration shell of the hyperthermophilic protein. These results indicate that *Sso AcP* at 80°C retains a small number of structured waters in the first hydration shell compared to *mt AcP* at 37°C, while the residence time of these waters result similar in the two systems, including functional MDHS in the active site. When analysing the second hydration shell, the peak height in the G(r) as well as the amount of MDHS were observed to be more similar in the two proteins, however, water dynamics were found to be significantly faster in *Sso AcP* at 80°C than in *mt AcP* at 37°C, thus reflecting the higher kinetic energy expected at these temperatures.

Taken together, our data identified some similar traits in the structural properties of waters residing in the first hydration shell of *mt AcP* and *Sso AcP*, and highlighted major differences in the dynamical behaviour of waters residing in regions further away from the protein surface. These latter waters likely are more influenced by the properties of the bulk, which induce faster relaxation in the second hydration shell of *Sso AcP* at 80°C. Thus, the concept of “corresponding states”, which indicates that homologous proteins from hypertermophilic and mesophilic organisms adopt similar structural dynamics at the respective physiological temperatures (Radestock and Gohlke, 2011; Fields et al., 2015), should be extended to the first hydration layer, which in our study was found to be the most similar region in the protein-water interface of *mt AcP* and *Sso AcP*.

## Data Availability

The raw data supporting the conclusion of this article will be made available by the authors, without undue reservation.
