# Supplementary material for: Thermal tuning of protein hydration in a hyperthermophilic enzyme
Source: Front Mol Biosci. 2022 Nov 28;9:1037445. doi: 10.3389/fmolb.2022.1037445 (PMC9742426; doi:10.3389/fmolb.2022.1037445)
Supplement: Supplementary file 1 [file DataSheet1.PDF]

*Supplementary Material*

# Thermal Tuning of Protein Hydration in a Hyperthermophilic Enzyme

**Giuliana Fusco[1], Carmen Biancaniello[2], Michail D. Vrettas[2] and Alfonso De Simone[2,*]**

[1]Department of Chemistry, University of Cambridge, Lensfield Road, CB2 1EW, Cambridge UK

[2]Department of Pharmacy, University of Naples "Federico II", via Montesano 49, 80131, Naples Italy

*** Correspondence:**
Alfonso De Simone
alfonso.desimone@unina.it

Figures S1-S6

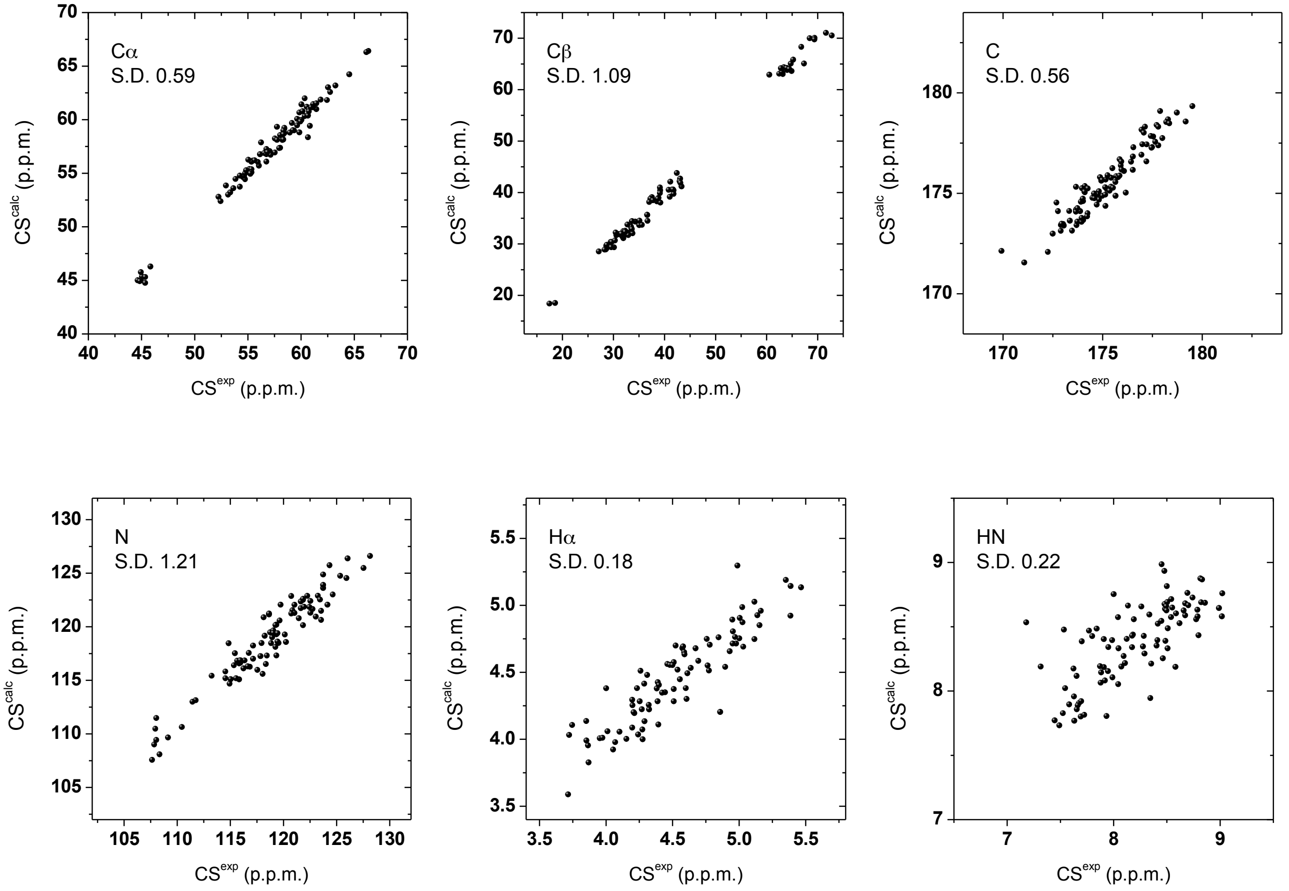


**Figure S1. Analysis of the CS of *mt AcP* at 37 °C.** Comparison between experimental CS and those calculated on the structures from the MD trajectory. The calculations were made using the SPARTA+ program (Shen & Bax, 2010, J Biomol NMR 48, 13-22). For each atom type, the error of the calculated CS results within the standard error of SPARTA+.

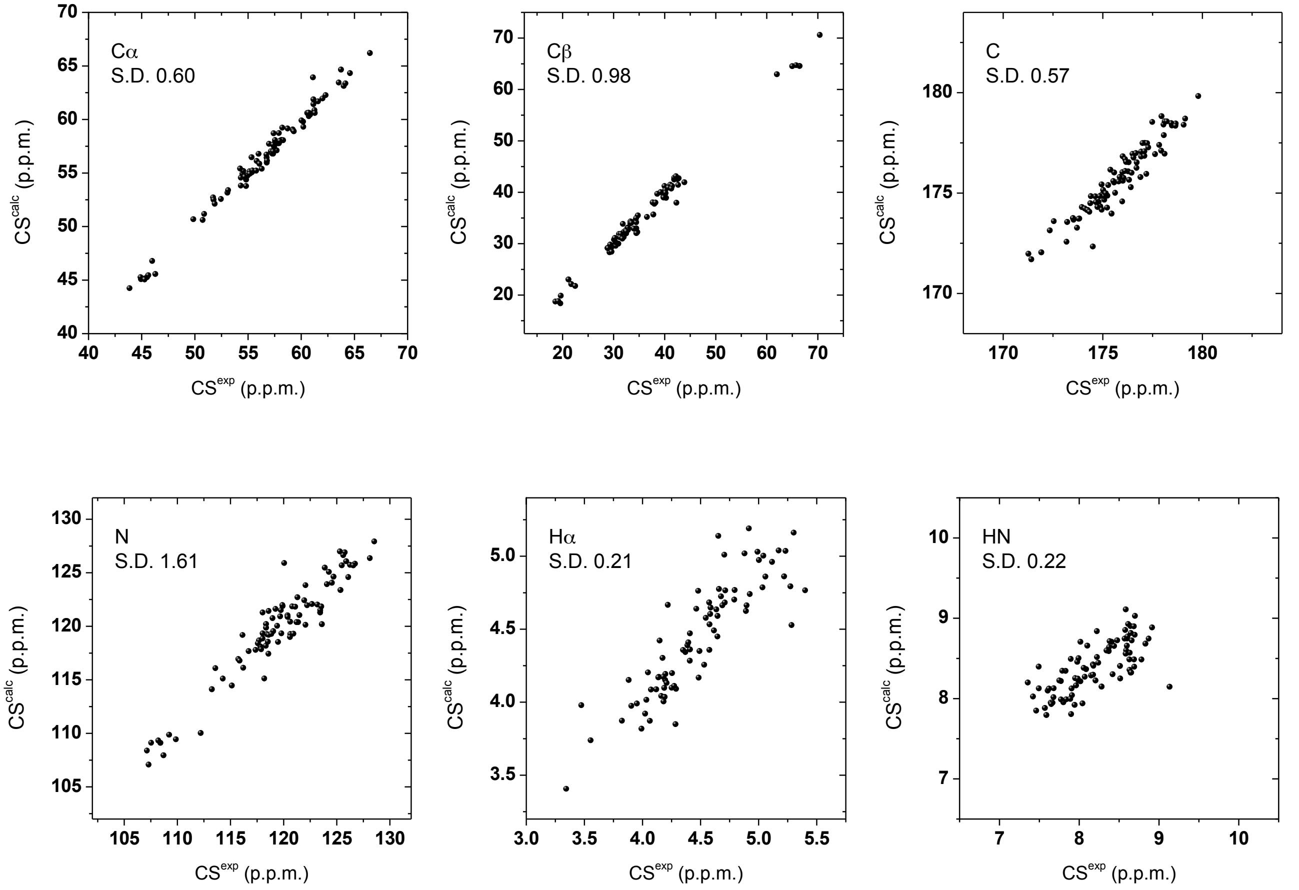


**Figure S2. Analysis of the CS of *Sso AcP* at 80 °C.** Comparison between experimental CS and those calculated on the structures from the MD trajectory. The calculations were made using the SPARTA+ program (Shen & Bax, 2010, J Biomol NMR 48, 13-22). For each atom type, the error of the calculated CS results within the standard error of SPARTA+.

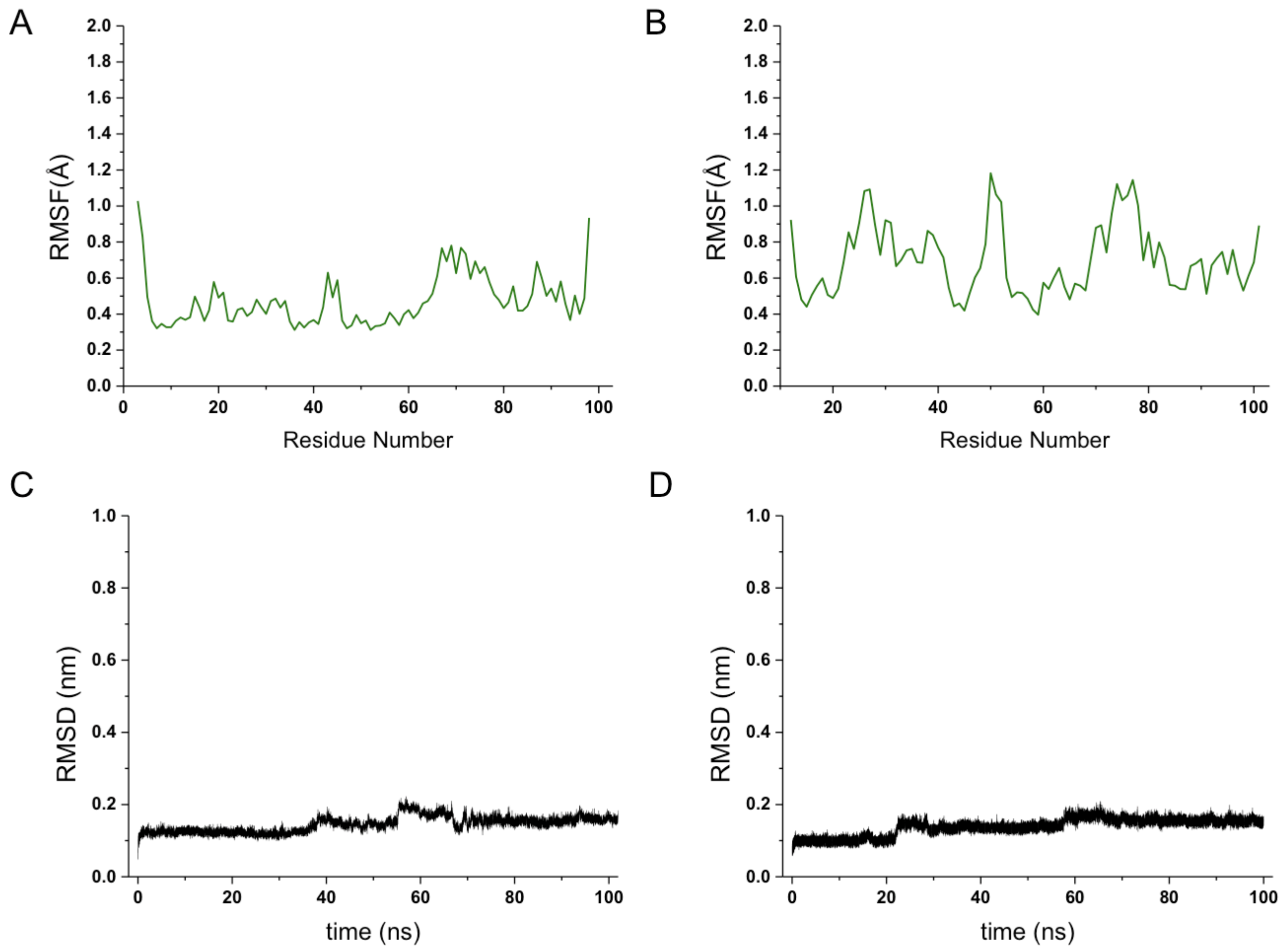


**Figure S3. RMSF and RMSD values in the simulations.** A-B) RMSF values of the simulations of *mt AcP* at 37 °C (A) and *Sso AcP* at 80 °C (B) calculated using Cα atoms. C-D) RMSD values from the starting experimental structures in the simulations of *mt AcP* at 37 °C (C) and *Sso AcP* at 80 °C (D) calculated using Cα atoms of non-loop regions of the proteins.

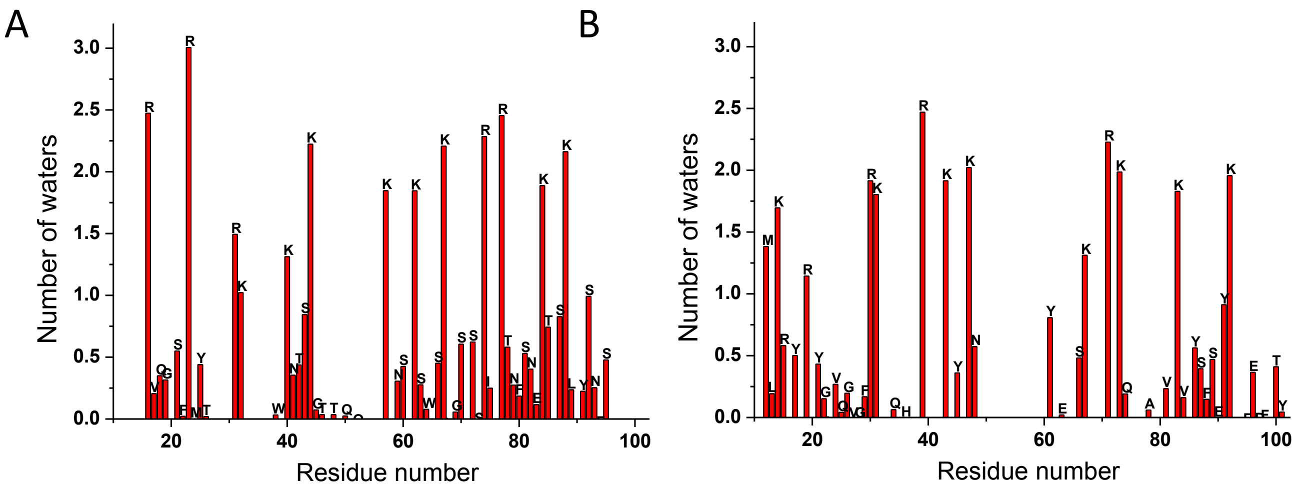


**Figure S4. Average number of waters surrounding the protein residues.** These values were calculated by calculating the distance between the oxygen atoms of waters and the protein atoms. For each frame of the simulations, a water molecule was attributed to a given protein residue if its minimum distance with any of the atoms of the residue was lower than 2.0 Å. The average values of the number of waters surrounding each protein residue in the restrained-MD trajectories are reported for *mt AcP* at 37 °C (A) and *Sso AcP* at 80 °C (B). The average numbers of waters surrounding the residues of *mt AcP* at 37 °C and *Sso AcP* at 80 °C resulted respectively 0.53 and 0.43.

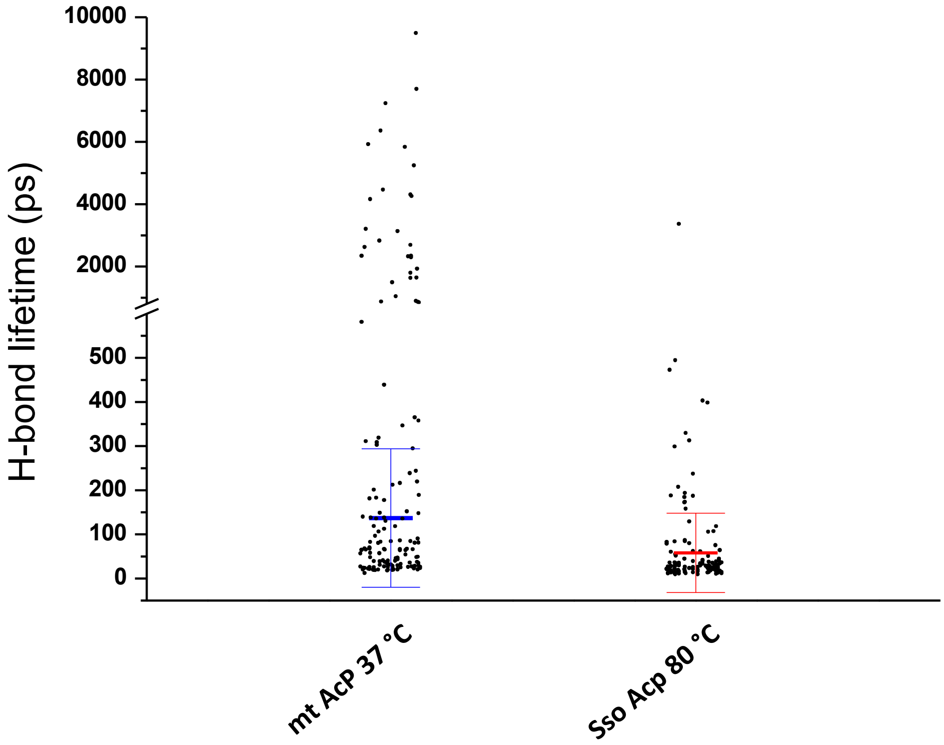


**Figure S5. Dynamic properties of protein-waters hydrogen bonds.** Lifetime of the H-bonds established between main chain and side chain groups of the proteins and the surrounding water molecules. This lifetime was calculated using the autocorrelation function, fitted with a single exponential decay model. Scatter plots of the residence times are reported for *mt AcP* at 37 °C and *Sso AcP* at 80 °C. In the mesophilic protein, the average lifetimes resulted 137.9 (± 190.7) ps and 58.7 (± 90.1) ps for *mt AcP* at 37 °C and *Sso AcP* at 80 °C, respectively. These average values were calculated by excluding superstructured H-bonds (designated as those featuring a lifetime higher than 1.0 ns).

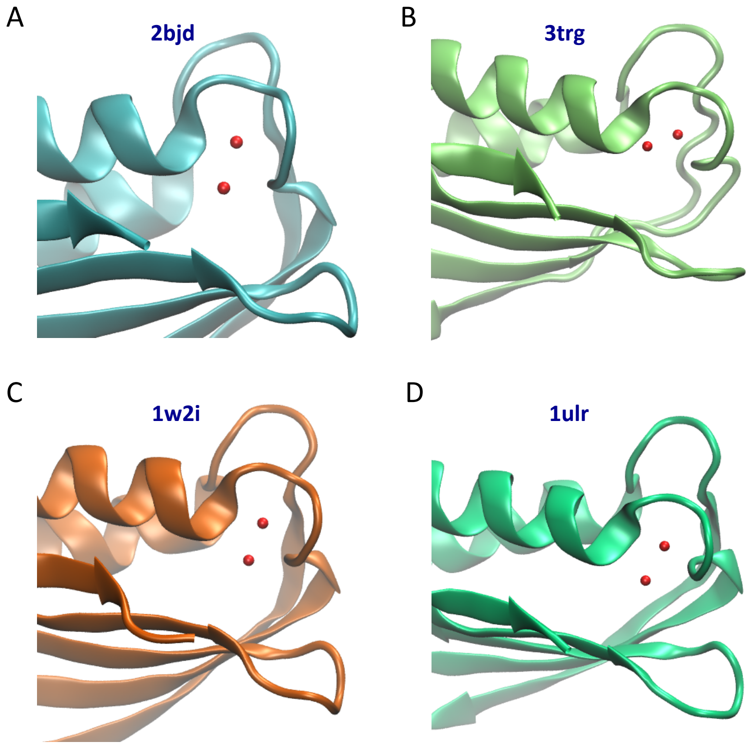


**Figure S6. Conserved hydration sites in crystal structures of AcP proteins from different organisms.** These waters were found to be conserved also in the CS-restrained MD simulations of *mt AcP* and *Sso AcP*. The figure shows the structures of AcP from *Sulfolobus solfataricus* (A), *Coxiella burnetii* (B), *Pyrococcus horikoshii* (C) and the putative AcP from *Thermus thermophilus* HB8 (D). PDB codes of the structures of these AcP are reported in each panel.
